# Update on Histological Reporting Changes in Neuroendocrine Neoplasms

**DOI:** 10.1007/s11912-021-01062-6

**Published:** 2021-04-14

**Authors:** Konstantin Bräutigam, Antonio Rodriguez-Calero, Corina Kim-Fuchs, Attila Kollár, Roman Trepp, Ilaria Marinoni, Aurel Perren

**Affiliations:** 1 Institute of Pathology, University of Bern, Murtenstrasse 31, 3008 Bern, Switzerland; 2 Department of Visceral Surgery and Medicine, Inselspital Bern University Hospital, University of Bern, Bern, Switzerland; 3 Department of Medical Oncology, Inselspital, Bern University Hospital, University of Bern, Bern, Switzerland; 4 Department of Diabetes, Endocrinology, Nutritional Medicine and Metabolism, Inselspital, Bern University Hospital and University of Bern, Bern, Switzerland

**Keywords:** Neuroendocrine tumour, NET, Classification, Reporting, NEN, Pathology

## Abstract

**Purpose of Review:**

Classification and nomenclature of neuroendocrine neoplasms (NEN) have frequently changed over the last years. These changes reflect both increasing knowledge and international standardisation.

**Recent Findings:**

The most recent changes in the Gastro-Entero-Pancreatic system induced the concept of well-differentiated NET with high proliferation rate (NET G3), explaining partially the heterogeneity of G3 NEN. Even if the nomenclature in pulmonary NEN is still different, the terms ‘carcinoid’ and ‘atypical carcinoid’ are widely overlapping with NET G1 and NET G2. Molecular data shows an additional heterogeneity both in well-differentiated NET and poorly differentiated NEC. However, no studies are available demonstrating clinical usefulness yet.

**Summary:**

The heterogeneity of NEN regarding the organ of origin, differentiation and molecular subtypes make development of personalised therapy a challenge needing more international and interdisciplinary collaborations and clinical trials allowing stratification according to biological subgroups.

## Introduction

### Historical Aspects

Neuroendocrine neoplasms (NENs) with an incidence of 7/100,000 per year [1] are rare and account for 3% of all cancers [2]. These 3% of cancers are furthermore distributed across the entire body, come in ‘low-grade’ and ‘high-grade’ flavours and therefore include a plethora of diagnoses. The incidence of NEN is highest in the lung and gastroenteropancreatic (GEP) system; therefore, nomenclature of these systems is often used in the rest of the body in analogy. From the description of the carcinoid tumour (of the ileum) by Oberndorfer in 1907 [3], the nomenclature has been developed by pathologists, specialised in specific organs and located in different countries, leading to a wide range of different names for similar tumours. These differences made comparison of studies and clinical trials very difficult. The concept to discriminate benign NEN from borderline and malignant NEN has additionally led to an uncertainty of what to register in individual cancer registries and if yes, how to register, leading to incomplete population based data. Over the last years, major efforts have led to a standardisation of nomenclature worldwide including the acceptance of the term neuroendocrine neoplasms (NENs), but there are still two mainstays of nomenclatures used across organs, the ‘GEP-NEN’ and the ‘lung carcinoid’ system. A convergence of these two systems is a strategic aim of future World Health Organisation (WHO) classifications [4••]. The following sections will focus on major classification changes of these neoplasms, underlining the progress of understanding them. During the same time, the level of details of pathological reports has dramatically increased as in other tumour entities, and synoptic reports are becoming more important tools to support pathologists, but also to allow a further use of the report data for clinical judgement, scientific projects and cancer reporting/registers [5, 6].

### NEN Classification Changes

**Fig. 1:**
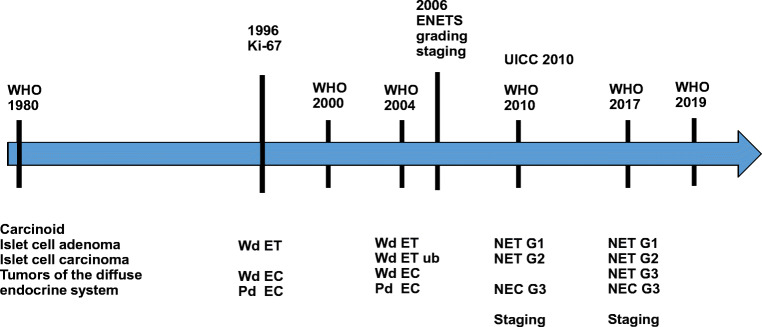
Classification of NEN, timeline since 1980. ENETS: European Neuroendocrine Tumor Society. NEC neuroendocrine carcinoma, NEN neuroendocrine neoplasm(s), NET neuroendocrine tumour, Pd EC poorly differentiated endocrine carcinoma, UICC Union Internationale Contre le Cancer, Wd EC well-differentiated endocrine carcinoma, Wd ET ub well-differentiated endocrine tumour uncertain behaviour, WHO World Health Organisation

There were not many changes of nomenclature before 2000: The WHO classification of 1980 used the terms islet cell adenoma/carcinoma, carcinoid and tumours of the diffuse endocrine system for pancreatic neuroendocrine tumours, depending on the cell of origin. The difference between ‘adenoma’ and ‘carcinoma’ was based on absence or presence of metastases. The first reports of risk assessment in NET by measuring proliferation using the cell cycle marker Ki-67 were published for Lung NET by Costes et al [7], followed by Pelosi et al. in pancreatic NET in 1996 [8]. In the WHO 2000 classification, the term well-differentiated endocrine tumour (ET) was introduced to replace the many different names based on putative cells of origin. The WHO 2004 classification did take account for different risks of progression; therefore, ETs were subdivided into ET of benign behaviour, ET of uncertain behaviour (increased Ki-67 and/or size and/or invasion) and well-differentiated endocrine carcinomas (presence of metastases). This concept of ‘uncertain behaviour’ did never find acceptance in the USA where this nomenclature change was not accepted, and still, many other terms were used. The European Neuroendocrine Tumor Society (ENETS) allowed in interdisciplinary consensus meetings in Frascati, Italy, to propose a system of grading based on proliferation (both mitotic index and Ki-67 index) as well as a TNM staging system. This proposition implied that all NETs bear some risk of metastases, however with important risk groups. Finally, the black-and-white separation between benign and malignant was gone, without needing to state it explicitly. A further relevant step of international standardisation in the GEP-NEN system was achieved with the WHO 2010 classification. On both sides of the Atlantic, the terms well-differentiated neuroendocrine tumour (NET) and poorly differentiated neuroendocrine carcinoma (NEC) were introduced, as was the overarching concept of neuroendocrine neoplasia (NEN) for the sum of the two biologically/genetically non-related entities. From this point of time onwards, diagnosis of the tumour entity (NET vs. NEC) was finally separated from grading and staging, as for other carcinomas. The Union Internationale Contre le cancer (UICC) and the American Joint Committee on Cancer (AJCC) largely followed the ENETS proposal and introduced a staging system for NET in the 7th edition [9]. Based on the different biology, for NEC, the staging of adenocarcinomas was and still is to be applied. Only after this separation it became increasingly apparent clinically, that not all NEN G3 are the same. A subgroup of pancreatic NEN (PanNEN) G3 is characterised by similar driver mutations as NET, and these seem to respond less to platinum-based therapy, and median survival is longer [10]. The separation of NET G3 from NEC G3 was defined in the pancreas first in the WHO 2017 classification and adapted to the entire GEP-system in the most recent WHO 2019 classification [10] (Fig. 1). This process of classification development was frequently driven by the pancreas, as most of the translational research activities focus on this site.

In contrast to all these changes in the GEP system, the classification of NEN of bronchopulmonary origin remained unchanged. The separation between typical carcinoid, atypical carcinoid and small cell/large cell neuroendocrine carcinoma is still used, still based on mitotic count and necrosis, and a clinically useful staging system for carcinoids and atypical carcinoids is still to be defined. The actual classifications of GEP-NEN and pulmonary NEN are summarised in Table 1.

**Table 1:** Gastroenteropancreatic Neuroendocrine Neoplasia (GEP-NEN, WHO 2019) vs. nomenclature of lung NEN (WHO 2015): The comparison of the two organ systems reveals that definition criteria of well-differentiated NET G1 and typical carcinoid of the lung as well as well-differentiated NET G2 and atypical carcinoid are similar but not identic. Necrosis has no significant relevance in GEP-NEN. More aggressive tumours, i.e. NEC and SCLC, have a wide mutational spectrum, with similar mutations in GEP- and lung NEN

GEP-NEN (WHO 2019) [11]				Lung NEN (WHO 2015) [12]		
	*Defining features*				*Defining features*	
Precursor lesions (e.g. neuroendocrine (micro)adenoma, neuroendocrine cell hyperplasia)	Histomorphology, size			Precursor lesions (e.g. DIPNECH, tumourlets)	Histomorphology, size	
Well-differentiated NET	*Grade* G1 G2 G3	*Mitotic Count (2mm*^*2*^*)* <2 2 to 20	*Ki67 Index (%)* <3 3 to 20 >20	Typical Carcinoid Atypical Carcinoid	*Necrosis* none none or focal	*Mitotic Count (2mm*^*2*^*)* 0 to 1 2 to 10
Poorly differentiated NEC	G3	>20 >20	>20	SCLC Large Cell NEC	Additional driver mutations (e.g. TP53, RB1)	>10 >10
MiNEN	Grading of both components	At least 30% of each		Combined carcinoma	At least 10% of each	

*DIPNECH* diffuse idiopathic pulmonary neuroendocrine cell hyperplasia, *MiNEN* mixed neuroendocrine-non-neuroendocrine neoplasm, *NEC* neuroendocrine carcinoma, *NET* neuroendocrine tumour, *SCLC* small cell lung cancer

### Evolution of Reporting Standards

While a decade ago tumour entity and distance to resection margins represented the main content of pathology reports, nowadays, increasing histopathological details are needed for optimal clinical treatment. Minimum diagnostic requirements defined by the ENETS-standard of care guidelines comprise definition and immunohistochemical confirmation of neuroendocrine phenotype, differentiation, grading and staging of the neoplasm [13]. As most of the translational research is performed in PanNEN, a focus on this entity is given in the following sections.

### Biomarkers

#### Diagnostic

In order to better differentiate and stratify NEN, several biomarkers have gained clinical relevance in the field of NEN over the last years (Table 2). First of all, neuroendocrine markers including Synaptophysin and Chromogranin A define the neuroendocrine phenotype [19]. CD56 is of minor relevance due to a lack of specificity. In order to estimate biological aggression, the mitotic count (per mm^2^) and the proliferation index Ki67 are important markers [20, 21]. Ki67 should be assessed in hotspots [4••] and preferably in manual or automatic counting as ‘eyeballing’ is not reliable [22, 23].

**Table 2:** Diagnostic, prognostic and predictive biomarkers

	Pancreas	Ileum	Lung	Other	NEC	All NEN
Diagnostic						
NEN diagnosis						Synaptophysin/chromogranin-A
NET vs NEC	DAXX/ATRX [14], MEN1		MEN1		p53, RB1, p16	
Organ of origin	Islet-1 [15], pancreatic hormones	CDX-2, Serotonin	TTF-1		none	
Prognostic						Ki-67
	CA9, microvessel density [16], DAXX/ATRX loss, CK19/c-Kit [17], epigenetic groups, cell of origin [18]	Loss of Chr. 18	MEN1			
Predictive	MGMT?			SSTR expression?	RB1 loss?	

*CA9* carboanhydrase 9, *Chr.* chromosome, *CK19* Cytokeratin 19, *c-KIT* tyrosine-protein kinase kit, *DAXX/ATRX* α-thalassemia mental retardation syndrome X-linked protein/ death-domain–associated protein, *MEN1* multiple endocrine neoplasia type 1, *MGMT* O^6^-methylguanine DNA methyltransferase, *NEC* neuroendocrine carcinoma, *NEN* neuroendocrine neoplasia, *RB1* retinoblastoma protein 1, *SSTR* Somatostatin receptor(s), *TTF-1* thyroid transcription factor-1

In the setting of functional NEN, detection of hormones may be of importance [24, 25]. Especially in the setting of multiple neuroendocrine tumours, such as in the Multiple Endocrine Neoplasia Type 1 (MEN1) syndrome, expression analysis of glucagon, insulin, gastrin and pancreatic polypeptide allows identification of which one is the tumour responsible for a hormonal syndrome.

In the context of metastatic NET of unknown primary, the set of transcription factors Islet-1, CDX2 and *Thyroid* transcription factor-1 may point towards a primary in the pancreas, small intestine or lung/thyroid respectively [15, 26].

Also, Somatostatin receptor 2A (SSTR2A) is a useful marker in NEN. SSTR2A is usually expressed in well-differentiated NEN and seems to be of prognostic value [27–29]. Furthermore, its expression has direct clinical impact as it might guide to peptide receptor radionuclide therapy (PRRT), mostly in metastatic or locally advanced, unresectable NEN [30]. Most of the approved high-energy radiopharmaceuticals have good receptor affinity for the SSTR2 receptor [31].

Mainly for the gastrointestinal tract, the differentiation between NET and NEC has improved due to new molecular-genetic insights. In NEC, the most aggressive forms of NEN, p53 and retinoblastoma protein 1 (RB1) have turned out to be important biomarkers. Aberrant p53 expression (TP53 inactivation) and the loss of RB are designated as features of pancreatic and gastrointestinal NEC [32–34]. RB protein and p53 expression can be investigated by immunohistochemistry. In addition, NECs share mutations (i.e. KRAS; SMAD4 in the pancreas, BRAF and K-Ras in the colon) with respective adenocarcinomas.

#### Prognostic

The most frequently used prognostic biomarker is Ki-67, which is the basis for grading of NEN. For pancreatic NET, many other prognostic biomarkers have been described to characterise more aggressive PanNET correlating to hypoxia [16], and stemness [17]; however, they have never found their way to clinical use. In recent years, alternative lengthening of telomeres (ALT) and loss of DAXX (death-domain associated protein) or ATRX (α-thalassemia mental retardation syndrome X-linked protein) were shown consistently to be associated with adverse outcome in resected PanNET [14, 35•, 36]. Loss of expression of either proteins correlates with higher tumour stage and grade [37].

In PanNET, an association of inflammatory features with prognosis becomes more evident. In pancreatic NEN, PD-L1 expression was associated with higher tumour grade [38], and strong expression was seen mostly in G3 NET. Cai et al. [39] describe the correlation of tumour-associated macrophages (TAMs) with reduced disease-free survival.

Several prognostic biomarkers have been described on the epigenetic level (see “Epigenetics”).

#### Predictive

While there is a series of approved drugs for treatment of metastasised NET, there is no predictive marker available. In particular, for tyrosin kinase receptor and mTOR inhibitors, a reliable clinical response prediction is missing. The expression of somatostatin-receptors, usually measured by molecular imaging (DOTA-SSA-PET/CT), is both prognostic and needed for somatostatin-receptor–targeted therapy. Regarding response prediction, SSTR2 expression is the best established factor in clinical routine as it might be indicative for treatment response with somatostatin analogues [40, 41].

#### Synoptic Reporting

Synoptic reporting is a standardised reporting format initially as defined by national societies such as by the College of American Pathologists (CAP) [42] or the Royal College of Pathologists (RCP) [43]. Synoptic reports consist of essential reporting elements as defined by national or international expert panels, which are to be reported in a reproducible value-like format. The International Collaboration on Cancer Reporting (ICCR) was founded by major pathology organisations from around the world for international standardisation. Regular updates are planned in a well-defined framework and aligned with WHO classification updates of tumours. While ICCR is still focusing on the most frequent cancer types, synoptic reports for NEN are available and regularly updated by CAP and RCP. In a structured way, synoptic reports for NEN begin with the TNM classification and grading. Tumour grading is further explained in a separate paragraph subdivided into Ki67 labelling index and mitotic count according to WHO guidelines. Later tumour invasion and resection margins are specified.

Synoptic reports do not exclude to report data points in addition to required data elements; therefore, in-house adaptations are feasible. It has been shown repeatedly that synoptic reports increase the completeness of pathology reports [44] potentially leading to better oncological treatment.

### Molecular Classifications

#### Epigenetics

Epigenetic changes are DNA modification, which do not affect the DNA sequence. They comprise DNA methylation, histone modifications or posttranscriptional control by microRNAs [45, 46]. In the last decade, the influence of epigenetic factors has been described in small intestinal NET, lung NET and PanNET [18, 33]. Notably, the majority of PanNET present with mutation in *MEN1*, *DAXX* and *ATRX*, which all encode for proteins involved in epigenetic regulation.

Epigenetic features, such as histone marks, signatures of super-enhancers as well as similarities of DNA methylation patterns to normal α- and β-cells have revealed at least three groups of PanNET with distinct cell of origin, clinical features and genetic background. Early-stage tumours, with MEN1 mutation, show a clear α-like epigenetic features and favourable outcome while benign insulinoma resemble β-cells. Tumours mutated in *DAXX* or *ATRX* showed an intermediate epigenetic profile but retaining α-like features. Intermediate ADM tumours have a poor prognosis [18, 47].

#### Mutational Profiles

The mutational landscape of PanNET, lung NET and ileal NET is well established. All well-differentiated NETs show a very low mutational burden. PanNET shows the highest rate of detectable driver mutations, mainly belonging to genes involved in DNA damage repair (5%), chromatin modification (40%), altered telomere length (40%) and mTOR signalling (15%) [48••]. In contrast, only <10% of ileal NET show recurring driver mutations. Besides *CKN1B*, many ileal NETs seem to carry private mutations. Lung NETs also have mutations in epigenetic modifiers such as *ARID1A* and others [49]. Alcala et al. have coined the term ‘supra-carcinoids’ [50•]. Supra-carcinoids seem to be a link between carcinoids and more aggressive large cell carcinomas, as their histopathology is similar to carcinoids, but the molecular phenotype the one of large cell NEC [50•]. Consensus in lung NEN nomenclature has not been reached so far.

The mutational spectrum is different in poorly differentiated NEC, where frequent mutations of *TP53*, inactivation of *RB1* and mutations of adenocarcinomas of the respective organ such as *K-Ras*, *BRAF* are found [51].

#### Transcriptomic Subgroups

Transcriptomic studies in mouse and human PanNET could reveal three subtypes: ‘well-differentiated islet tumours’ or ‘insulinoma-like’ (IT), ‘intermediate’ and ‘metastasis-like primary’ [52]. The MLP subtype is more aggressive and characterised by features of hypoxia, stemness and an immune-related phenotype of viral mimicry [48••, 52–54]. Due to a lack of potential predictive value, none of these molecular markers have found application into clinical practice.

Poorly differentiated pulmonary NEC, small cell lung cancer and large cell neuroendocrine lung cancer show three subgroups with different transcriptomic profiles of NOTCH signalling, neuroendocrine profile, metabolism and cell cycle [55]. On a mutational level, group 1 is characterised by STK11/KEAP1 mutations, groups 2 (mainly large cell NEC) and 3 (mainly small cell NEC) are characterised by RB mutation or loss of expression and P16 (CDKN2A) mutation. There seems to be some association of absence of RB1 mutation/loss and better response to chemotherapy including platinum and gemcitabine or taxanes [56].

## Conclusions

The histopathological classification has developed stepwise to the concepts of NEN of different grades, with different mutational spectra depending on organ site. Rarely, NEN can also have high proliferation rates and are classified as NET G3 due to morphological, clinical and genetic similarities to well-differentiated NET of low proliferative activity.

Poorly differentiated NECs are separated due to a different biology, morphology and genetics closer to adenocarcinomas of the respective organs than to NET.

Molecular data demonstrates that these morphological groups are still heterogeneous based on mutational, epigenetic and transcriptomic (also proteomic and metabolomic) features. This biological heterogeneity is not taken into account by actual treatment options, as many NETs are treated in very similar ways, and NECs are treated mainly in analogy to data from lung tumours.

While the morphological classification seems to be at a state of maturity, further efforts are needed to benefit from the feasible and established molecular classifications with respect to therapy indication and response. Therefore, it is foreseeable that in addition to actual classification, reporting of biomarkers or molecular subgroups will be of increasing importance.
